# Pedagogical resilience reconstruction in the AI era: a longitudinal reflexive thematic analysis of teacher reflective journals

**DOI:** 10.3389/fpsyg.2026.1910640

**Published:** 2026-10-26

**Authors:** Hu Gao, Xiaona Liu, Chang Liu

**Affiliations:** 1 General Education Department, Shaanxi Vocational Academy of Art, Xi'an, Shaanxi, China; 2 Teacher Education Department, Hengshui University, Hengshui, Hebei, China; 3 Foreign Languages Department, Hengshui University, Hengshui, Hebei, China

**Keywords:** digital literacy, educational equity, generative artificial intelligence, interdisciplinary teaching, pedagogical resilience, reflexive thematic analysis

## Abstract

**Objective:**

Generative artificial intelligence (GenAI) lowers the threshold for producing work, not necessarily for learning. This study examines how an instructor kept an interdisciplinary AI course teachable for first-year art students, where that effort reached its limits, and how the instructor interpreted students' engagement with AI tools.

**Methods:**

The study is a longitudinal practitioner self-study of a 12-week course taught in autumn 2025 at a higher vocational college in northwestern China. The data are the instructor's own reflective journals: structured entries written during the semester for Weeks 1–7 and retrospective accounts of Weeks 8–12 written after the semester ended. The two strata are reported separately. Reflexive thematic analysis produced 40 evidence anchors, five themes, one cross-cutting thread, and one central organizing theme, with a full audit trail.

**Results:**

The instructor's repairs (translating content into everyday and discipline-specific tasks, working around broken or restricted lab equipment, and demonstrating on the teacher's computer) kept the course running. However, as content moved toward frontier tools that required paid accounts, computing resources, and prior AI experience, teaching drifted toward demonstration (“the more cutting-edge something is, the more it can only be demonstrated”). Across the semester the instructor recorded a recurring pattern, here termed a teacher-perceived *low-threshold illusion*, in which students appeared, in the instructor's account, to treat seeing, bookmarking, or one-click generation as equivalent to being able to do. The instructor associated this pattern with three co-occurring conditions: natural-language prompting and one-click generation, demonstration-only teaching, and a final assessment that students could complete without having attended. Participation could be high while, in the instructor's judgement, students gained little.

**Conclusion:**

Pedagogical resilience reconstruction in AI teaching is bounded. Keeping an AI course running did not by itself make learning visible: the journals record a tension between engagement, product completion, and evidence of learning, not an established failure of learning. In-course repair reached limits shaped by access conditions and an inherited assessment format, and reconstruction of the course took place at the scale of the semester. We present pedagogical resilience reconstruction as an emerging construct for multi-case testing.

## 1 Introduction

Generative artificial intelligence (GenAI) has made it easy to produce a slide deck, an image, or a short video from a single instruction. What it has not made easy is learning to judge, revise, and reuse what such tools produce. Experimental work suggests that learners supported by ChatGPT can reach higher short-term task scores without corresponding gains in knowledge transfer, and may disengage from the metacognitive work of checking and revising (Fan et al., 2025). Broader reviews describe GenAI as both a powerful learning resource and a potential crutch (Yan et al., 2024; Zhai et al., 2024). For teachers, the problem therefore extends beyond introducing tools to students. It becomes a question of how to keep learning visible when completing a product no longer shows that learning has taken place.

This problem is especially acute in interdisciplinary AI courses for non-technical majors. Such courses often have no settled curriculum, run on institutional infrastructure that lags behind the tools being taught, and are taught by teachers whose own disciplinary training lies elsewhere (Tan et al., 2024). Much of the literature on teachers and AI relies on cross-sectional surveys or interviews about perceptions and readiness (Kim, 2024; Zhai, 2025). These designs cannot show how a teacher's responses change week by week as a course unfolds, nor where those responses stop working.

Research on teacher resilience has largely framed resilience as a teacher's psychological capacity to sustain commitment and effectiveness under pressure (Gu and Day, 2007; Beltman et al., 2011), including emotional adaptation in the AI era (Liu and Chang, 2024). This study takes a complementary angle. It asks what happens to the *pedagogical arrangement* (tasks, examples, language, tools, and assessment) when a teacher tries to keep an AI course teachable under unstable conditions. It also asks which parts of that arrangement lie beyond the teacher's reach.

We report a longitudinal practitioner self-study of one 12-week course, drawing on the instructor's own reflective journals. Three research questions guided the analysis:

- **RQ1:** How was pedagogical resilience reconstructed across W1–W12 in interdisciplinary AI teaching, and what turning points and limits are visible in the teacher's journals?
- **RQ2:** How did the teacher interpret students' engagement with AI tools across the course, and with which classroom conditions were these interpretations associated?
- **RQ3:** Under what boundary conditions did AI-supported tasks support or constrain educational equity and digital literacy, understood here as access and learning opportunity?

The study makes one central contribution. It shows that in-course pedagogical repair can keep a course operable while the teacher remains unable to see whether substantial learning is taking place, and it locates the limits of that repair in access conditions and assessment design, not only in the teacher's individual capacity. We interpret this pattern through the construct of *pedagogical resilience reconstruction*, specify its boundaries, and present it as an emerging analytical construct rather than an established one.

## 2 Conceptual background and analytical framework

### 2.1 From teacher resilience to pedagogical resilience

Teacher resilience research has shown that resilience is not a fixed trait but a relational, context-dependent process of sustaining commitment and effectiveness (Gu and Day, 2007; Beltman et al., 2011). Related work examines how teachers adapt instruction in real time. *Adaptive teaching* refers to adjusting instruction to learners' needs as they become visible (Corno, 2008; Parsons et al., 2018). *Pedagogical improvisation* describes creative, in-the-moment departures from a plan within a structured lesson (Sawyer, 2011). *Adaptive expertise* is the individual capacity to apply knowledge flexibly to novel problems (Hatano and Inagaki, 1986). *Teacher noticing* describes how teachers attend to and interpret classroom events (Sherin et al., 2011). *Reflective practice* describes how practitioners learn from experience by reflecting in and on action (Schön, 1983). *Instructional redesign*, including design-based research, refers to the planned, iterative refinement of a learning design and its principles across implementations (Design-Based Research Collective, 2003; McKenney and Reeves, 2018).

Each of these constructs is useful, and together they cover much of what teachers do when a lesson does not go to plan or a course is revised. None of them, however, combines three properties that the present case requires. First, the object of analysis is the pedagogical arrangement (tasks, examples, language, tools, and assessment) rather than the teacher's commitment, knowledge, attention, or expertise. Second, the construct distinguishes two time scales: in-course repair, which keeps an arrangement running while teaching is under way, and semester-scale reconstruction, which redesigns the arrangement between offerings. Adaptive teaching and improvisation concern the first. Instructional redesign concerns the second, but it typically starts from design principles to be tested rather than from repairs made under pressure. Third, the construct attends to the limits of in-course repair and to the conditions, largely set outside the classroom, that shape those limits. Table 1 sets out these boundaries. Given the single-case nature of the evidence, we treat *pedagogical resilience reconstruction* as an emerging analytical construct whose usefulness must be tested in further cases.

**Table 1:** Construct boundaries between pedagogical resilience reconstruction and related constructs.

Construct	Unit of analysis	Typical time scale	What it explains	What it does not foreground
Teacher resilience (Gu and Day, 2007; Beltman et al., 2011)	The teacher (commitment, well-being, effectiveness)	Career, year	How teachers sustain themselves under pressure	Changes in the pedagogical arrangement itself
Adaptive teaching (Corno, 2008; Parsons et al., 2018)	Teacher–learner interaction	Lesson, unit	How instruction is adjusted to learner needs	Constraints that make adjustment impossible
Pedagogical improvisation (Sawyer, 2011)	Classroom interaction within a plan	Moment, lesson	Creative departures from a lesson plan	Courses without a stable plan to depart from
Adaptive expertise (Hatano and Inagaki, 1986)	Individual expertise	Long-term development	Flexible application of knowledge to novel problems	Structural conditions under which expertise cannot act
Teacher noticing (Sherin et al., 2011)	The teacher's attention and reasoning	Moment, lesson	What teachers perceive in classroom events and how they interpret it	What happens to the arrangement when noticing cannot be acted upon
Reflective practice (Schön, 1983)	The practitioner's learning from experience	Action, then after action	How practitioners reframe problems through reflection	Constraints on acting on what reflection reveals
Instructional redesign/design-based research (Design-Based Research Collective, 2003; McKenney and Reeves, 2018)	The learning design and its principles	Iterative cycles across implementations	How a design is refined through planned iteration	Unplanned repair under constraints before a stable design exists
Pedagogical resilience reconstruction (this study)	The pedagogical arrangement (tasks, examples, language, tools, assessment)	Week to semester	How an arrangement is kept operable under unstable conditions, and where in-course repair reaches its limits	Teachers' inner states; causal effects on student outcomes

### 2.2 Access, skills, and the second-level digital divide

Digital-divide research has long argued that equal access to devices does not produce equal benefit. Differences in skills and in patterns of use form a “second-level” divide (Hargittai, 2002), and inequality increasingly shifts from access to usage (van Deursen and van Dijk, 2014). Work on GenAI in education makes a parallel point: AI alone will not democratize education (Bulathwela et al., 2024), and its equity effects depend on access, literacy, pedagogy, and governance (Cong-Lem et al., 2025; Gabriel, 2024). Equity can refer to access, participation, learning opportunity, or outcomes. This study makes claims only in two of these senses, for which the data provide evidence: *access* (devices, accounts, computing resources) and *learning opportunity* (whether students could take part in hands-on learning at all). Differences in participation are described but not interpreted as inequity, and outcome equity is not addressed.

### 2.3 Seeing vs. doing: learner-side risks as sensitizing concepts

Cognitive research has documented that people can mistake the fluency of processing for knowing (Oppenheimer, 2008), and that learners' judgments of competence during study can be inflated relative to later performance (Bjork, 1994; Koriat and Bjork, 2005). Research on GenAI adds evidence of cognitive offloading (Gerlich, 2025) and reduced metacognitive engagement when revision is not required (Fan et al., 2025). In this study these concepts are used only as *sensitizing* resources. No student-level psychological state was measured. Interpretations of students in this paper are the instructor's interpretations, recorded in the journals, and are reported as such.

### 2.4 Other sensitizing lenses

Two further lenses informed, but did not structure, the analysis. TPACK and its AI extensions describe the teacher's task of matching technological, pedagogical, and content knowledge (Mishra and Koehler, 2006; Mishra et al., 2023; Ning et al., 2024). They help explain why an English teacher assigned to an AI course had to rebuild what counted as “basic content”. Transformative learning (Mezirow, 1991) was used only to read the instructor's Week 6 change in frame. We do not claim that a full transformative cycle occurred.

## 3 Materials and methods

### 3.1 Design and positionality

The study is a longitudinal practitioner self-study (Pinnegar and Hamilton, 2009; Louden, 1991) analyzed through reflexive thematic analysis (Braun and Clarke, 2006, 2019, 2021). This is the primary design. The Context–Mechanism–Outcome (CMO) scheme was used only as a heuristic for sorting evidence anchors into conditions the instructor faced, responses the instructor made, and outcomes the instructor recorded. We do not adopt the assumptions of realist evaluation, and we do not claim process tracing in the causal sense.

The first author was the course instructor, the author of the journals, and the analyst. The instructor's disciplinary background is English language teaching. The instructor learned AI tools independently and was assigned to teach this course for the first time. This position gave the study direct access to week-by-week teaching decisions. It also means that what was noticed, recorded, selected as evidence, and theorized all passed through a single perspective. The reflexive implications are discussed in the Trustworthiness section below.

### 3.2 Setting

The course, “Information Technology and Artificial Intelligence Applications”, was a compulsory general-education course taught over 12 weeks in the first semester of the 2025–2026 academic year at a higher vocational college in northwestern China. It was taught to several classes of first-year students (approximately 90 in total) majoring in animation, digital media, calligraphy and painting, and arts and crafts. For animation and digital media students, computers were part of their professional work; for calligraphy and arts-and-crafts students, they largely were not. Table 2 lists the weekly topics.

**Table 2:** Weekly topics and data strata.

Week	Topic	AI tools named	Data stratum
W1	Buying a computer with AI assistance	Gamma (slides)	Contemporaneous, template
W2	File management, software installation, compression, online safety	Gamma	Contemporaneous, template
W3	Prompting strategies	Kimi, Doubao, Cherry Studio	Contemporaneous, template
W4	Word documents; separating format from content	Kimi, Markdown-to-Word converter	Contemporaneous, template
W5	Slides: from PowerPoint to templates to AI generation	Gamma, Kimi, Marp	Contemporaneous, template
W6	AI-assisted English learning	Duolingo, Doubao	Contemporaneous, template
W7	Note-taking tools and a “second brain”	Obsidian, Claude Code	Contemporaneous, template
W8	Spreadsheets with AI	(AI control of Excel)	Retrospective, free-form
W9	Micro-lesson production (image, music, video)	Multiple paid tools	Retrospective, free-form
W10	Workflows and agents	(demonstration and online videos)	Retrospective, free-form
W11	Major-specific AI agent use cases	Image generation	Retrospective, free-form
W12	Final-exam rehearsal (classical poem to image and video)	Mobile AI tools	Retrospective, free-form

### 3.3 Data

The data consist solely of the instructor's reflective journals, in two strata that differ in when and how they were written (Table 2).

*Stratum 1: contemporaneous structured journals (Weeks 1–7)*. During the semester, the instructor completed a seven-part template after each week's teaching: activities and AI tools; interdisciplinary adaptation; sustainability elements; teacher growth; classroom effects and student feedback; challenges; and improvement plans. The sustainability section was left blank or near-blank in most weeks and was therefore not analyzed as a theme.

*Stratum 2: retrospective accounts (Weeks 8–12)*. The template was not completed for Weeks 8–12. After the semester, the instructor wrote short free-form accounts of these weeks from memory, without reference to contemporaneous records. These accounts are necessarily shaped by hindsight. At least one passage explicitly reports a decision taken after the semester (removing the workflow unit). We therefore report Stratum 2 separately and do not treat differences between the two strata as evidence of change over time.

During the semester, the instructor pasted several journal entries into general-purpose GenAI chatbots (ChatGPT and DeepSeek; see Generative AI statement) and received interpretive commentary on them. Only text written by the instructor was analyzed; all AI-generated commentary was excluded.

No student work, classroom observation records, or student interviews were analyzed. Students appear in the data only as the instructor described them, collectively and without identifying information. The single student utterance in the data (Week 1) is the instructor's report of an anonymous remark.

### 3.4 Analysis

The analysis followed the six phases of reflexive thematic analysis (Braun and Clarke, 2006, 2021). In the first phase the instructor-researcher re-read the full corpus in the original Chinese. In the second phase, *evidence anchors* were extracted: bounded passages describing a teaching decision, a constraint, a student behavior as observed by the instructor, or an instructor judgment. Each anchor was assigned an initial code and a CMO heuristic label. This produced 40 anchors (32 contemporaneous, 8 retrospective). All 40 were labeled C (14), M (17), or O (9). CMO labels mark an anchor's analytic role only; implementation status was coded separately as recorded practice or observation, a plan recorded in the template's improvement section, or a post-semester decision. A provisional M label on a planned response (EA09, EA23, EA28, EA32) indicates its analytic role and does not establish that the response was implemented; EA36 records a post-semester decision. In the third to fifth phases, codes were clustered into candidate themes, reviewed against the anchors, and defined. The result was five themes, one cross-cutting thread that recurs across themes, and one central organizing theme. Each anchor was assigned to exactly one primary theme (Table 3). Table 4 summarizes the coding counts. In the sixth phase, quotations were translated into English for reporting. A generative AI tool assisted with extracting passages by line reference, checking counts, proposing initial codes and theme groupings, and translating quotations (see Generative AI statement); all codes, theme assignments, and translations were reviewed against the original Chinese journals by the first author.

**Table 3:** Themes, primary evidence anchors, and weeks.

Theme	Primary anchors (*n*)	Weeks	Stratum
A. Translation without a template, and the week 6 reframing	EA01, 03, 06, 13, 14, 15, 18, 19, 22, 24, 29 (11)	W1–W7	Contemporaneous
B. Linguistic scaffolding: English as a hidden entry key	EA11, 12, 26, 27 (4)	W3, W6	Contemporaneous
C. The drift to demonstration: from broken labs to paywalls	EA05, 08, 09, 17, 31, 32, 34, 36 (8)	W2, W4, W7, W9, W10	Both
D. Uneven relevance and engagement	EA02, 04, 07, 16, 21, 25, 28, 30, 37 (9)	W1, W2, W4–W7, W11	Both
E. Assessment and the visibility of learning	EA23, 38, 39 (3)	W5, W12	Both
Cross-cutting thread: the teacher-perceived low-threshold illusion	EA10, 20, 33, 35, 40 (5)	W3, W5, W8, W10, W12	Both
Total	40		

**Table 4:** Coding statistics.

Coding stage	Count
Evidence anchors	40 (contemporaneous W1–W7: 32; retrospective W8–W12: 8)
CMO heuristic: conditions (C)	14
CMO heuristic: responses/mechanisms (M)	17
CMO heuristic: outcomes (O)	9
Implementation status: planned responses (all labeled M)	4
Implementation status: post-semester decision	1
Themes	5
Cross-cutting thread	1
Central organizing theme	1

Table 5 illustrates the full chain from raw excerpt to central theme for two anchors. Supplementary Table 1 lists all 40 anchors with source file and line reference, the original Chinese text, an English translation, the initial code, the CMO label, and the theme assignment.

**Table 5:** Worked examples of the analytic chain.

Step	Example 1 (W3)	Example 2 (W10)
Raw excerpt (translated)	“Once a prompt was shown, they seemed to lose the motivation to try it themselves… with prompts they feel they already understand after seeing them once… or they simply bookmark the prompt methods to consult later.”	“The more cutting-edge something is, the more it can only be demonstrated… So there is an illusion that AI tools require no entry threshold… In actual hands-on operation, they did not even know where to paste an API key.”
Data stratum	Contemporaneous	Retrospective
Evidence anchor	EA10: students treat seeing a prompt as knowing it	EA35: frontier content can only be demonstrated; perceived absence of thresholds
Initial code	Seeing-as-knowing	Frontier–demonstration paradox; hidden threshold; perceived low threshold
Theme	Cross-cutting thread: teacher-perceived low-threshold illusion	Cross-cutting thread (primary); also informs Theme C
CMO heuristic	O (instructor-recorded outcome)	C (condition), also recorded as outcome
Interpretive status	Instructor's interpretation of observed behavior; no student-level measurement	Instructor's interpretation, written after the semester
Central organizing theme	Pedagogical resilience reconstruction and its limits: in-course repair (demonstration, attention-catching examples) drew students' interest, while the conditions that let students skip doing remained in place	Same; the limit of in-course repair is set by access conditions

### 3.5 Trustworthiness and reflexivity

Four features of the design bear on trustworthiness, and we address each directly.

#### 3.5.1 Single perspective

The instructor was teacher, grader, journal writer, and analyst, and this shaped the evidence at four points. *What was noticed:* the journals record what one teacher attended to, so students who were learning quietly, and problems the teacher did not see, are absent. *What was recorded:* the seven-part template for Weeks 1–7 directed attention to particular dimensions, such as adaptation to students' majors and the challenges encountered, and this shaped what was written; the Week 8–12 accounts are brief and unstructured. *What was selected as evidence:* selection was made by the same person, so to make it checkable, Supplementary Table 1 lists every selected passage with its source line and original text. *What was later theorized:* see the next two paragraphs. Throughout, every interpretation of students is reported as the instructor's interpretation, and analytic claims are restricted to what the instructor recorded doing and observing.

#### 3.5.2 Two data strata

The contemporaneous entries are structured, dense, and written close to the events. The retrospective accounts are brief, unstructured, and written months later. Themes that appear mainly in the contemporaneous stratum, such as translation work (Theme A), may seem to fade in later weeks simply because the retrospective accounts are shorter and do not follow the template. We therefore describe the course as a *retrospectively constructed trajectory* and not as a directly observed developmental sequence.

#### 3.5.3 Hindsight and prior interpretation

The retrospective accounts of Weeks 8–12 were written after the semester by the same person who analyzed the data, and after an initial interpretation of the course had already been formed. They may therefore have been shaped both by later knowledge, including the post-semester redesign they mention, and by that interpretation. We read them as the instructor's considered account of those weeks, not as a record of events. There is one check on this risk: the retrospective accounts did not confirm the initial interpretation. An earlier reading of the course as a progression from emergency substitution to structured iteration was not supported by them and was abandoned.

#### 3.5.4 Grading role and sampling

Because no student work was analyzed, the study makes no claims about the distribution of student performance and involves no sampling of assignments by quality. The instructor's grading role still matters, because the instructor's views of students' effort were formed partly while grading. This is one reason we do not interpret student behavior in psychological terms.

## 4 Results

The central organizing theme is *pedagogical resilience reconstruction and its limits*: the instructor's repair repertoire kept the course operable, but once content outran students' access, teaching drifted toward demonstration, and the instructor's records show a tension between engagement, product completion, and the visibility of learning. The five themes and the cross-cutting thread are summarized in Table 3, and Figure 1 maps when each appears in the two data strata.

**Figure 1:**
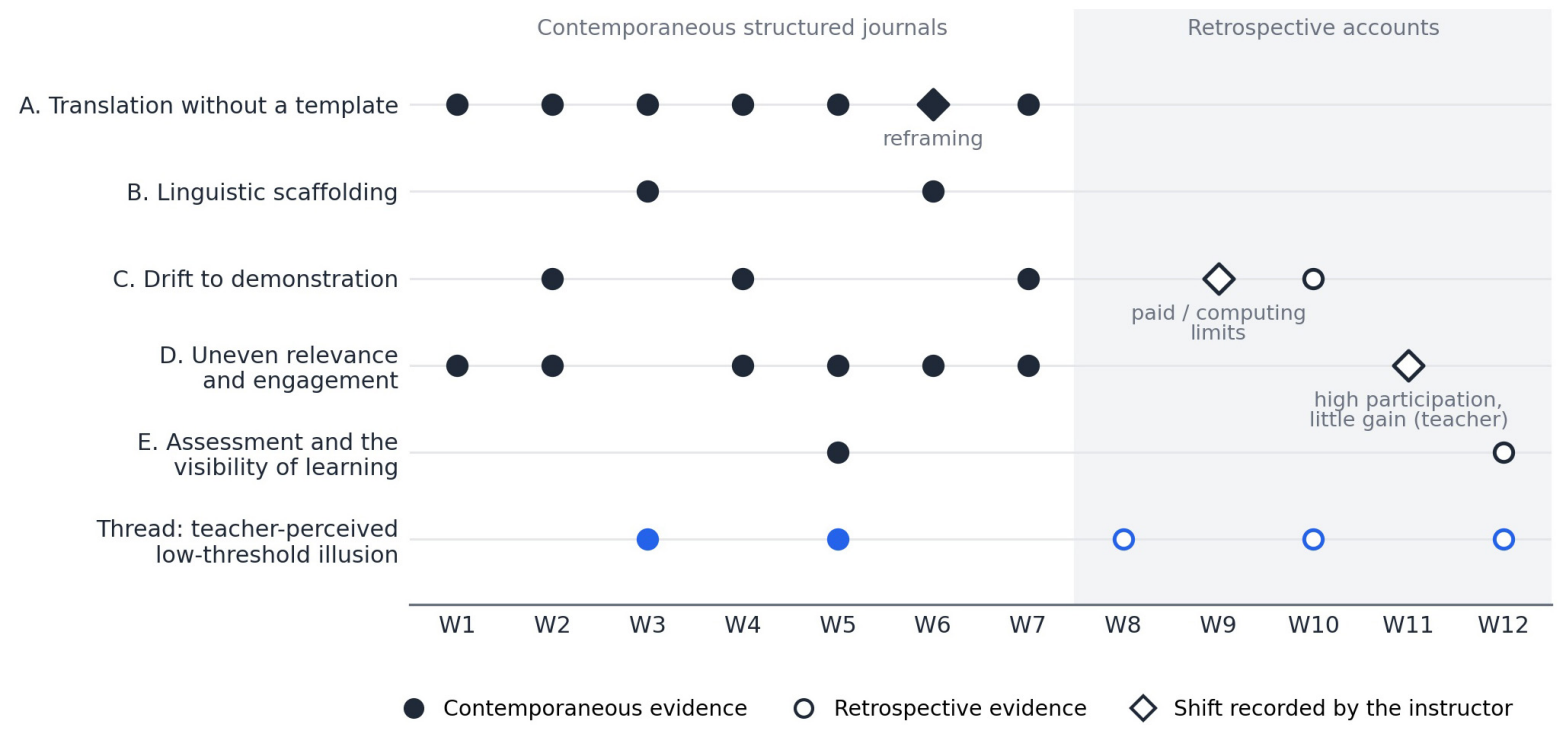
Qualitative temporal map of themes across Weeks 1–12. A marker indicates a week in which a theme has at least one primary evidence anchor (Supplementary Table 1). Filled markers denote contemporaneous and open markers retrospective evidence; the shaded band marks the retrospective accounts (W8–W12). Diamonds mark shifts recorded by the instructor: the Week 6 reframing (EA24), the turn to paid and computing limits in Week 9 (EA34), and high participation with little gain, in the instructor's judgement, in Week 11 (EA37). Emergence, persistence, and recurrence can be read from the sequence of markers. The map shows presence only; it does not represent intensity.

### 4.1 Theme A. Translation without a template, and the week 6 reframing

The instructor's first problem was not a shortage of tools but the absence of a course to teach. In the first week, the planned opening on the origins and principles of computing was dropped because “this content was outdated and did not fit the students' majors.” It was replaced by a scenario in which first-year students used AI to understand the specifications of a computer they might buy (EA01). By Week 2, the instructor recorded a more basic uncertainty:

“In English teaching we routinely explain how words are used, but in this course I do not yet know what the most basic knowledge is. I can only see what students cannot do during hands-on practice.” (W2, EA06)

The teaching repertoire the instructor brought from English also failed to transfer. In English, “preparing some major-related videos and vocabulary is enough to connect with students' disciplines”. In a computing course, students “from majors that do not use computers show low motivation” (W1, EA03). Much of the instructor's work over the following weeks was translation: redesigning scattered online material written for already-motivated learners into classroom-ready tasks (W3, EA13), preparing different prompt examples for different majors (W3, EA14), introducing the principle of separating format from content through AI-assisted document writing (W4, EA15), sequencing slide-making from PowerPoint to templates to AI generation while insisting that slides “cannot be entirely handed over to AI” (W5, EA19), and setting a self-introduction as the slide task to make it personally relevant (W5, EA22). The instructor also closed gaps in their own knowledge (“my computing knowledge is not systematic; I specifically reviewed the relevant content,” W4, EA18).

Week 6 was a turning point in how the instructor framed this work. The topic, AI-assisted English learning, returned the instructor to their home discipline, yet the entry records a deliberate distance from the English-teacher role:

“English was my previous teaching subject, so this class felt easy and familiar. Yet I did not actually teach English content… I need more empathy to understand students' difficulties in learning English, rather than the mode of an English teacher who places many demands on students.” (W6, EA24)

In teacher-noticing terms (Sherin et al., 2011), attention shifted from whether students met requirements to where they met barriers. We read this as a change in the instructor's frame consistent with the early stages of transformative learning (Mezirow, 1991). The data do not show that change being consolidated into a redesigned course during the semester: the Week 6 plan for “better tiering for students with different needs” (EA28) does not reappear in later entries. In Week 7, the instructor made a different kind of judgment. Knowledge-management tools were “cutting-edge and practical” and “should be introduced to students”, although “only a minority of students sense the importance of this content” (EA29). This judgment about what was worth teaching anticipates the tension described in Theme C.

### 4.2 Theme B. Linguistic scaffolding: English as a hidden entry key

The most concrete strand of the instructor's translation work concerned language. In Week 3 the instructor noted that English prompts produced better results, but that this advantage could not easily be passed on:

“Using English prompts does give better results, but for now the benefit is limited to my own use; getting students to use English prompts is difficult—this is, after all, not an English class. I mostly introduced the thinking behind prompts, and that thinking comes from English-language news and tutorials.” (W3, EA11)

Two things are visible here. First, the instructor's access to current AI practice depended on English-language sources, so the instructor acted as a translator between that material and a Chinese-language classroom. Second, what the instructor passed on was not the English prompts themselves but “the thinking behind” them, rendered in forms students could use. The instructor was also unsure where this knowledge belonged: prompting “is neither Chinese nor English, nor quite computing. It is more like a kind of logic that sits beneath these subjects” (W3, EA12). In scaffolding terms (van de Pol et al., 2010), the instructor diagnosed a language barrier and responded by changing the form of the input, not by requiring students to cross the barrier.

In Week 6 the same resource was turned the other way. AI prompts were used to generate “vocabulary and exercises better matched to students' majors,” with some majors emphasizing oral expression and others exam preparation (EA26). The instructor also recorded that many students “do not recognize the importance of English” and lacked the motivation to learn it (EA27). Taken together, the entries suggest that English functioned as a hidden entry key to current AI knowledge. Students with stronger English could presumably reach such sources directly, whereas students without it depended on the instructor's translation; the journals do not record this directly, so we treat it as an inference. We return to this as a learning-opportunity condition under RQ3.

The remark that “this is, after all, not an English class” also connects this theme to the Week 6 reframing (Theme A). In Week 3 the instructor set aside the English-teacher role by declining to require English. In Week 6, back in the home discipline, the instructor explicitly rejected “the mode of an English teacher who places many demands on students” (EA24). In both weeks, the instructor's English expertise was turned from a subject to be taught into a resource for translation. The Week 3 entry also names the cost of that translation work: online prompt material was “not systematic” and written for already-motivated readers, and turning it into “material that can be used directly in a university classroom requires redesign” (EA13).

### 4.3 Theme C. The drift to demonstration: from broken labs to paywalls

Physical infrastructure was the first constraint. In Week 2, “many computers in the school's lab are broken, and new hardware will not arrive until next year.” The instructor added that explaining through slides alone “can hardly work, because these problems only appear when actually operating a computer” (EA08). The instructor's improvement plan was to “add some online videos, combine them with my own demonstration, and then add more hands-on practice” (EA09); later entries record demonstration and videos being used (W3, W7), but not additional hands-on practice. In Week 4, lab computers lacked the necessary tools and ran an old version of Word, so the instructor used online converters and their own laptop (EA17). In Week 7, lab restrictions meant that the note-taking and AI software “could only be demonstrated on the teacher's computer” (EA31). The planned response was to switch to “more integrated, easier-to-use domestic software” (EA32).

In the retrospective accounts of Weeks 9 and 10, the constraint changed character: it became economic and computational rather than physical. Producing a micro-lesson required several AI tools, “all of which are paid; free accounts do not have enough quota. In the end it could only remain at the demonstration stage” (W9, EA34). In Week 10, workflows and agents were taught “mainly by demonstration,” supplemented by online videos. The instructor summarized the pattern:

“This is a contradiction of AI courses: the more cutting-edge something is, the more it can only be demonstrated, because it needs hardware, paid accounts—computing power, in other words.” (W10, EA35).

We call this the *frontier–demonstration paradox*. The workarounds recorded in Weeks 2–7 (online tools and the teacher's own laptop, W4) and the planned switch to easier domestic software (W7) addressed missing or restricted equipment; none of them addressed paywalls or computing requirements. Demonstration thus changed from a stopgap into the default mode for the most advanced content. The instructor's structural response came after the semester: “Right after the semester ended, I removed this workflow unit and replaced it with newer, more automated agent content… I must keep learning and update teaching content at least every semester” (EA36). Because this passage was written retrospectively, we treat it as the instructor's account of a post-semester decision. It shows that redesign of the arrangement took place at the scale of the semester, outside the course itself.

Online safety formed a smaller strand of this theme. In Week 2 the instructor observed that students “often opened a fake AI website and started chatting before they had even found the official site of the AI application” (EA05). Even the most basic form of access, reaching the genuine tool, could not be assumed.

### 4.4 Theme D. Uneven relevance and engagement

Professional relevance shaped engagement from the first week. Calligraphy and arts-and-crafts students “do not need computers for their majors… so the lesson held limited appeal for them” (W1, EA02). Art students “currently have few occasions to use Word” (W4, EA16). The one student utterance in the data expresses early skepticism rather than fear: “I have used Doubao and it doesn't feel very smart” (W1, EA04). In Week 2, “most students kept their heads down in silence or looked at their phones” when asked about AI (EA07). By Week 6 the instructor had observed a divided pattern of use: students “often sit at two extremes: not using AI at all or relying on it excessively”, with enthusiasm varying “by major and by their further-study and employment prospects” (EA25).

The instructor linked part of the engagement problem to short-form video: it “easily takes away students' classroom attention, making them lose patience with slides” (W5, EA21). This is the only entry of its kind. We treat it as a contextual hypothesis held by the instructor and consistent with research linking extended screen time to continuous partial attention (Fırat, 2025). It is not an explanatory mechanism established by these data.

The retrospective accounts add two distinct observations. In Week 9, one class's “attendance had clearly dropped” (EA34). In Week 11, when the instructor presented major-specific AI use cases, such as image generation for clothing and make-up suggestions, “classroom participation was high and the atmosphere good, but I feel they did not gain much from it” (EA37). The instructor thus separated engagement from learning. The records do not show that tailoring generally raised engagement: major-specific examples and tasks coexisted with average participation in Week 3 and low participation in Week 7, and only the Week 11 account records high participation. The journals document the instructor's concern that high engagement did not necessarily entail substantial learning; they do not establish that learning was absent.

### 4.5 Theme E. Assessment and the visibility of learning

In Week 5 the instructor noted that presenting slides “is a composite skill; students need to present on stage” (EA23). Producing a deck was not the same as communicating with it. The final assessment, described in the Week 12 account, raised the same issue more sharply. The exam had been designed by another teacher, and the instructor adapted its content within the same format: students generated images and a short video from a classical Chinese poem, a task that “can be done entirely on a phone”, and “students' completion rate was high” (EA38). The instructor then observed:

“Even students who had not attended a single class this semester could produce almost the same final video. Although the work of students who studied seriously was clearly better…” (W12, EA39)

The instructor's account points in two directions. Product completion provided limited evidence about learning, since students who had not attended could, in the instructor's view, produce similar videos; yet the instructor also judged that students who studied seriously produced better work. The records therefore show a tension between product completion and the visibility of learning, not an assessment that could not distinguish learning at all. In constructive-alignment terms (Biggs, 1996), the assessed task centered on producing an artifact rather than on the processes the course aimed to teach. Its format was designed by another teacher and was retained, with adapted content, in the instructor's account; the journals do not record whether the instructor could have changed the format.

### 4.6 Cross-cutting thread: the teacher-perceived low-threshold illusion

One pattern recurs across the semester and cuts across the themes above. In the instructor's own words, it is an “illusion” (*cuojue*, W10; *huanjue*, W12) that AI tools have no threshold. We present it as the instructor's interpretation of what students did, not as a measured student belief. Its evidence base is five anchors (Figure 2):

**Figure 2:**
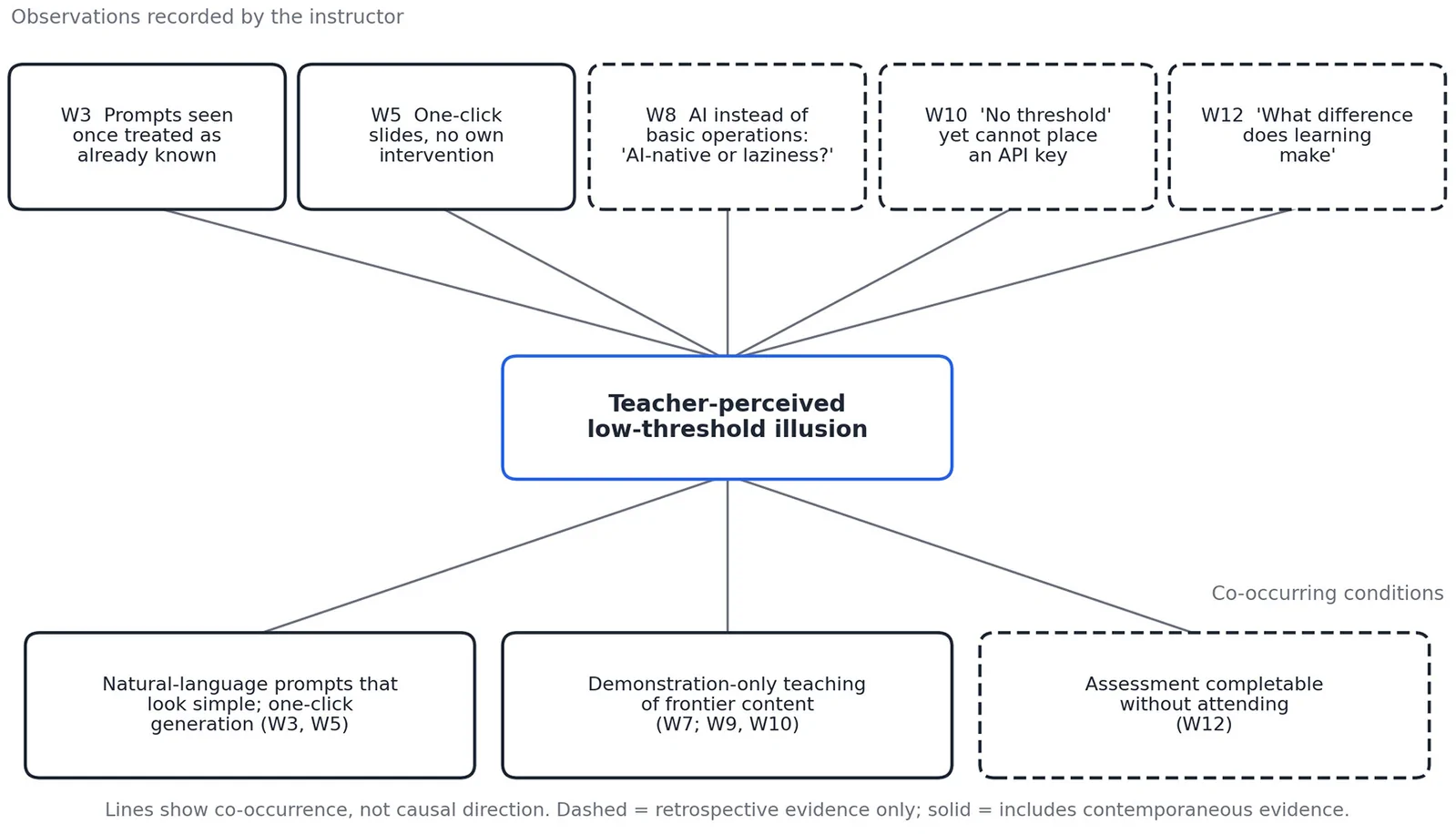
The teacher-perceived low-threshold illusion and three co-occurring classroom conditions. Lines indicate co-occurrence in the instructor's accounts, not causal direction. Solid outlines mark boxes supported at least partly by contemporaneous evidence; dashed outlines mark boxes supported by retrospective evidence only.

- **W3 (EA10, contemporaneous):** The instructor recorded that students were impressed by advanced prompts but did not adapt them. “With prompts they feel they already understand after seeing them once… or they simply bookmark the prompt methods.”
- **W5 (EA20, contemporaneous):** Students may “rely too heavily on one-click slide generation… without their own intervention, which ultimately leads to poor presentations.”
- **W8 (EA33, retrospective):** Faced with spreadsheet tasks they found difficult, students “chose to operate directly with AI and abandoned the traditional way. I do not know whether this is being truly ‘AI-native' or just laziness.”
- **W10 (EA35, retrospective):** “There is an illusion that AI tools require no entry threshold and that everyone can learn them easily. In actual hands-on operation, they did not even know where to paste an API key.” The instructor added that “prior AI experience is also an invisiblethreshold.”
- **W12 (EA40, retrospective):** The final assessment “deepened their illusion about AI's threshold. Some students felt that even without those techniques, what they made was passable—so what difference does learning make.”

Two features of this thread matter for interpretation. First, the instructor's own uncertainty is part of the data. The Week 8 entry explicitly leaves open whether bypassing traditional operations was an adaptive, “AI-native” choice or avoidance of effort. We preserve that ambiguity rather than resolving it with a psychological label. Second, the entries that link the pattern to the learning environment come mainly from the retrospective accounts (W10, W12), whereas an early contemporaneous entry also invokes students' motivation (W2). This is a contrast in emphasis between the two data strata, not a directly observed change over time. The illusion is associated with natural-language prompting that looks simple (W3) and one-click generation (W5), with demonstration as the only available mode for frontier content (W10), and with an assessment that could be completed without attending (W12). The pattern resembles documented illusions of competence during study (Bjork, 1994; Koriat and Bjork, 2005), but we use that literature only to name what the instructor described, not to claim that the same mechanism operated.

### 4.7 Central organizing theme and CMO-informed summary

Figure 3 brings the themes together. Read with the CMO heuristic, the *conditions* the instructor faced were the absence of a curricular template, uneven professional relevance across majors, language barriers, and access constraints that shifted from broken lab machines to paid accounts and computing requirements. The instructor's *responses* were a set of in-course repairs: translation into everyday and major-specific tasks, linguistic scaffolding, workarounds, and demonstration. The *outcomes* the instructor recorded were mixed. The course stayed operable, but frontier content drifted toward demonstration, the low-threshold illusion recurred in the instructor's accounts, participation was uneven, and the product-based final assessment provided limited evidence about learning. Reconstruction of the arrangement itself took place after the semester. The figure organizes the themes and does not represent a causal model.

**Figure 3:**
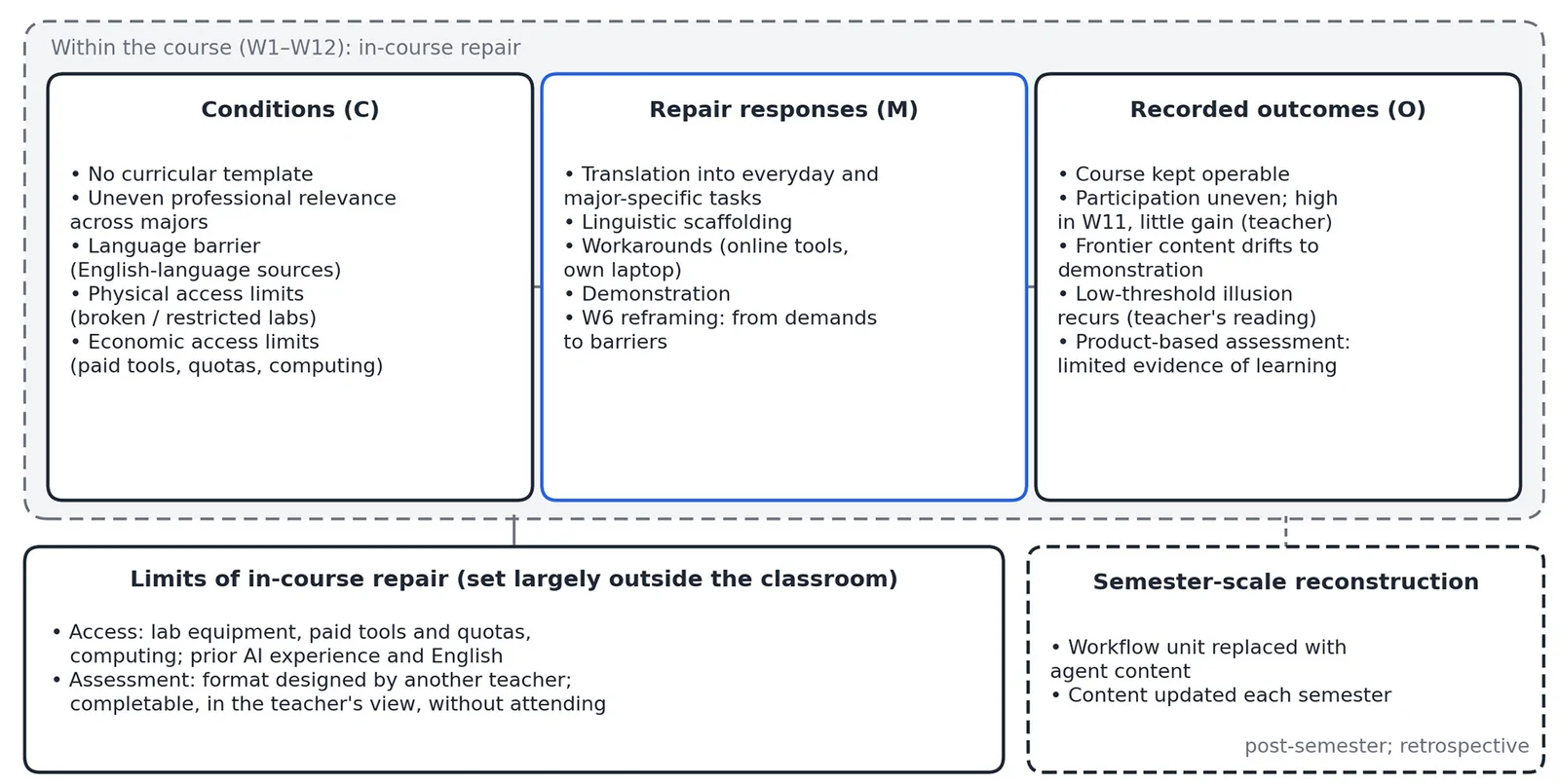
Thematic model of pedagogical resilience reconstruction and its limits. The three columns inside the shaded frame (in-course repair) follow the CMO heuristic used to organize the analysis; connectors indicate thematic grouping, not causal paths. The solid box below lists the limits of in-course repair, which were set largely outside the classroom. The dashed box shows semester-scale reconstruction, which is reported only in the retrospective account.

## 5 Discussion

### 5.1 RQ1: how pedagogical resilience was reconstructed over 12 weeks

Pedagogical resilience was reconstructed in two registers. Within the course, the contemporaneous journals for Weeks 1–7 record active repair work: translation, workarounds, major-specific tailoring, and demonstration that kept a course without a template running. The clearest turning point is Week 6, when the instructor's frame shifted from placing demands on students to understanding where they met barriers. In the retrospective accounts of Weeks 9–10, the same repertoire met constraints it could not route around: paid accounts, quotas, computing requirements, and the invisible threshold of prior AI experience. Demonstration changed from a stopgap into the default mode. In the second register, the substantive reconstruction of the pedagogical arrangement (replacing the workflow unit, committing to semester-by-semester updating) took place after the course had ended.

The trajectory is therefore not a linear rise from emergency response to structured iteration. It is better described as in-course repair that sustained operability, followed by a visible limit, followed by redesign at a longer time scale. Because Weeks 8–12 are retrospective, this is a retrospectively constructed trajectory rather than a directly observed developmental sequence. This echoes Rind and Qureshi (2026), whose interviews with teachers working with AI suggest that teacher agency persists but becomes conditional on institutional pressures and professional confidence. The present study adds a longitudinal view of where, within one course, that condition began to bind.

### 5.2 RQ2: how the teacher interpreted students

The two data strata differ in where they locate the source of students' difficulties. An early contemporaneous entry attributes low engagement partly to motivation and to the post-examination “freedom” of university life (W2), and the Week 6 entry turns toward the barriers students faced. The retrospective accounts of Weeks 10 and 12 name interfaces, demonstration-only teaching, and assessment design as conditions that sustained the low-threshold illusion. Because the later accounts were written after the semester, this is a retrospective contrast in emphasis, not a directly observed change in the teacher's thinking over time. What the data document is therefore not a student psychological mechanism but a teacher's account that, especially in retrospect, locates the problem in the design of the learning environment rather than in students alone.

If the illusion is sustained by the environment, it cannot be corrected by exhortation alone. Tasks in which seeing and doing visibly come apart can help: for example, students adapt a demonstrated prompt to a new case, or explain why an AI-generated slide works or fails. Such tasks require that students have hands-on access, and they require an assessment that rewards the process.

### 5.3 Alternative explanations

Four alternative explanations deserve attention. First, the trajectory may reflect the ordinary learning curve of teaching a new course for the first time. A learning curve would predict increasing stability over time. The later weeks instead show increasing reliance on demonstration and declining attendance in one class. This does not rule out a learning-curve explanation, since the instructor did learn and redesign. It does suggest that constraints distinctive to AI courses, such as rapidly changing tools, paywalled capabilities, and hidden prior-experience thresholds, shaped where that learning could be applied.

Second, the apparent change between the first and second halves of the course may be partly an artifact of the two data strata. The structured, contemporaneous entries for Weeks 1–7 invite detailed accounts of translation work. The brief retrospective accounts of Weeks 8–12 do not. The fading of Theme A after Week 7 (Figure 1) may reflect this difference in the data as much as any change in teaching. For this reason we do not interpret differences between the strata as change over time.

Third, declining engagement may reflect novelty decay rather than the conditions identified here. The data do not show uniform decline, however. Participation was recorded as high in Week 11, late in the course, even though the teacher judged that students gained little. High participation late in the course is not what novelty decay alone would predict.

Fourth, the behavior the teacher read as a low-threshold illusion may reflect students' effort-saving choices or rational responses to an assessment that did not reward process. The teacher's own Week 8 uncertainty (“AI-native” or “laziness”) and Week 12 account of the assessment are consistent with this reading. We therefore treat the illusion as the teacher's interpretation and treat the assessment design as one of its co-occurring conditions, not as a separate cause.

### 5.4 RQ3: boundary conditions for equity and digital literacy

We address the four senses of equity in turn. On *access*, the teacher recorded constraints of two kinds: broken or restricted laboratory machines (W2, W4, W7) and, in the retrospective accounts, paid tools, free-tier quotas, and computing requirements (W9, W10). The first concerned shared institutional equipment; the second could, in principle, differ between students who can and cannot obtain accounts outside class, but the journals do not record how students differed in this respect. On *participation*, the journals record uneven participation across majors (W1, W4, W6) and declining attendance in one class (W9), but they do not allow us to separate inequity from differences in interest or perceived relevance, so we describe these only as patterns of participation. On *learning opportunity*, the teacher identified two possible hidden entry conditions: prior AI experience, which the teacher called “an invisible threshold” (W10), and English, the language of the sources on which current AI practice depends (W3). These resemble the second-level digital divide described in the literature (Hargittai, 2002; van Deursen and van Dijk, 2014). The study identifies them as constraints named by the teacher; it does not establish their differential effects across student groups. If frontier content can only be demonstrated in class, such conditions may matter more, because practice then depends on resources that students bring with them; this remains a hypothesis for further research. On *outcome* equity we make no claim, because student outcomes were not analyzed. The paper's equity claims are therefore confined to access and learning opportunity. On digital literacy, Week 2 shows that even reaching the genuine tool could not be assumed.

Rind (20260 argues that inequities in access can create new cognitive and ethical burdens in AI-mediated teaching. The present case shows one concrete form such a burden can take for teachers: repeatedly re-translating inaccessible material into forms that some students can use.

### 5.5 Theoretical contribution 1: why “pedagogical resilience reconstruction” rather than “teacher resilience”

Teacher resilience usually refers to a teacher's capacity to sustain commitment and effectiveness under pressure (Gu and Day, 2007; Beltman et al., 2011). Pedagogical resilience, as used here, refers to the capacity of the pedagogical arrangement (tasks, examples, language, tools, and assessment) to remain operable and learnable under pressure. Table 1 distinguishes the construct from adaptive teaching, improvisation, adaptive expertise, teacher noticing, reflective practice, and instructional redesign. The case clarifies what the construct adds. Adaptive teaching and improvisation describe adjustments within a lesson or unit (Corno, 2008; Sawyer, 2011). This case shows those adjustments keeping the course operable, and it also shows where they reached their limits: frontier content could only be demonstrated, and the teacher could not see whether substantial learning was taking place. Beyond that point, the resilience of the arrangement depended on decisions made at a longer time scale and on conditions largely set outside the classroom, including laboratory equipment, the cost of tools, and an assessment format designed by another teacher. The construct thus directs attention to the *limits* of in-course adaptation and to the conditions that set them, which the related constructs do not foreground.

Two sensitizing lenses help interpret the case without adding further claims. TPACK (Mishra and Koehler, 2006; Mishra et al., 2023; Ning et al., 2024) helps explain why an English teacher assigned to an AI course had to rebuild what counted as basic content (W2) and why linguistic scaffolding became central (Theme B). The teacher's content knowledge of English became a resource for translating AI practice, not a subject to be taught. Transformative learning (Mezirow, 1991) helps read the Week 6 reframing as a change in the teacher's frame. We do not claim that a full transformative cycle was completed within the course.

### 5.6 “Reconstruction” and adaptive adjustment

The term reconstruction is used cautiously. Within the course, the evidence shows repair: the arrangement was kept running. Reconstruction in the stronger sense, a redesign of the arrangement itself, appears only in the teacher's account of the post-semester decision to replace the workflow unit and to update content every semester. This resembles the iterative cycles of design-based research (Design-Based Research Collective, 2003; McKenney and Reeves, 2018). It differs in that it did not begin from a design principle to be tested; the design principles emerged from the limits met in teaching. Whether the redesign improves learning in later offerings is an empirical question this study cannot answer.

### 5.7 Theoretical contribution 2: relocating the student-side problem

The second contribution concerns how the student-side problem is located. Much writing on GenAI in education frames problems such as overreliance or reduced verification as properties of learners (Zhai et al., 2024; Gerlich, 2025). In this case, a teacher whose early entry attributes low engagement partly to motivation associates the pattern of treating seeing as doing, especially in the retrospective accounts, with natural-language prompting and one-click generation, demonstration-only teaching, and assessment design. The teacher-perceived low-threshold illusion is therefore offered not as a new learner construct but as a description of how the pattern appeared to the person responsible for the learning environment.

### 5.8 Theoretical, methodological, and practical contributions

Methodologically, the study shows how a practitioner self-study can make its evidence base transparent. It separates contemporaneous from retrospective data, reports every evidence anchor with its original text, and keeps the teacher's interpretations of students distinct from claims about students.

Practically, three implications follow, each qualified by the single-case design. First, AI courses for non-technical students need institutional provision of accounts and computing resources, or deliberate selection of tools that can be used hands-on. Otherwise the most current content will tend to be taught by demonstration. Second, assessment in such courses needs to capture process, for example through drafts, revision notes, or explanations of choices. A product-only task that can be completed without attending may reinforce the low-threshold illusion. Third, because course reconstruction happens at the scale of the semester, teachers need structured time and support to rebuild AI courses between offerings, not only in-class adaptability.

### 5.9 Limitations and future research

The study has four principal limitations. First, it rests on one teacher's journals in one course. Its contribution is analytic rather than statistical generalization, and the construct must be tested across teachers, institutions, and disciplines. Second, the data contain no student perspectives. All statements about students are the teacher's interpretations, and future research should use student interviews, stimulated recall, or process data to test them. Third, Weeks 8–12 are retrospective accounts written after the semester. They are shorter, unstructured, and vulnerable to hindsight, so differences between Weeks 1–7 and Weeks 8–12 cannot be read as change over time. Fourth, the teacher used GenAI chatbots (ChatGPT and DeepSeek) to comment on some journal entries during the semester. Although the AI commentary was excluded from analysis, it may have influenced what the teacher attended to in later entries. Future work should also follow the redesigned course into later semesters to examine whether semester-scale reconstruction changes what students can do.

## 6 Conclusion

This study followed one teacher's attempt to keep an interdisciplinary AI course teachable for first-year art students. The teacher's repairs kept the course running. As content outran students' access, teaching drifted toward demonstration, and the teacher's records show a tension between engagement, product completion, and the visibility of learning: participation could be high while, in the teacher's judgement, students gained little, and the final product showed learning only partly. The teacher recorded a teacher-perceived low-threshold illusion, and the teacher's accounts, especially the retrospective ones, link it to natural-language prompting and one-click generation, demonstration-only teaching, and assessment design rather than to students alone. The reconstruction of the course itself happened after the semester. Pedagogical resilience reconstruction in the AI era is therefore bounded: keeping an AI course running does not by itself make learning visible, and making it visible depends partly on conditions set outside the classroom.

## Data Availability

The instructor-authored journal text analysed in this study is provided in the original Chinese, with English translations of all evidence anchors, in Supplementary Table 1. Further instructor-authored journal text is available from the corresponding author on reasonable request.

## Supplementary material

**Supplementary Table 1**

Audit trail of the 40 evidence anchors, listing for each anchor the source file and line, the original Chinese text, the English translation, the initial code, the CMO heuristic label, the implementation status, and the primary theme.
