# Supplementary material for: Pedagogical resilience reconstruction in the AI era: a longitudinal reflexive thematic analysis of teacher reflective journals
Source: Front Psychol. 2026 Oct 26;17:1910640. doi: 10.3389/fpsyg.2026.1910640 (PMC13745475; doi:10.3389/fpsyg.2026.1910640)
Supplement: Supplementary file 1 [file Table_1.docx]

# 1 Supplementary Table S1. Full audit trail: 40 evidence anchors

Source files: R2 = 教学反思记录-2.md; R1 = 教学反思记录.md; R8 = AI 教学反思日志week 8-12.md. Stratum: C = contemporaneous structured journal (W1–W7); R = retrospective account written after the semester (W8–W12). Original text is the instructor’s verbatim Chinese (full line(s) cited; English translation excerpts the analytically relevant part). CMO labels mark analytic role only; implementation status is coded separately in the Status column, and a provisional M label on a planned response does not establish that it was implemented. 〔……〕 marks one omitted clause that evaluates the institution and carries no analytic content; 〔sic〕 marks a typographical error in the original.

| ID | Week | Source (file:line) | Stratum | Original text (Chinese, verbatim) | English translation (excerpt) | Initial code | CMO | Status | Theme |
| --- | --- | --- | --- | --- | --- | --- | --- | --- | --- |
| EA01 | W1 | R2:57 | C | 最大的调整是原来的计算机课程设计是要从计算机的起源和基本原理讲起的，但是这些内容过于陈旧，与学生的专业也不符合。要从一个新的贴近生活的切入点开始介绍计算机的基础知识和 AI，所以我设计了一个大一新生买电脑的场景，在看不懂专业名词的情况下，如何借助 AI 工具，从了解自己地需求到了解术语，最终购买适合自己的新电脑 | The original computer course was designed to begin with the origins and basic principles of computing, but this content was outdated and did not fit the students’ majors… so I designed a scenario in which first-year students buy a computer. | Everyday-scenario translation | M | Recorded practice or observation | A. Translation without a template |
| EA02 | W1 | R2:16,26 | C | 对于书画和工美专业的学生，他们的专业不需要使用电脑，他们没有购买电脑的需求，因此这次课程对他们来说吸引力一般，但是介绍如何与 AI 聊天帮助自己理解一些电脑的概念，如内存，CPU 等在手机中也会出现的概念，可以帮助他们更好的了解信息技术的常识。 因此像书画专业的同学，兴趣点和参与度并不高 | Calligraphy and arts-and-crafts students do not need computers for their majors… so the lesson held limited appeal for them. / Students such as those in calligraphy showed low interest and participation. | Differential professional relevance | C | Recorded practice or observation | D. Uneven relevance and engagement |
| EA03 | W1 | R2:41 | C | 意识到了计算机和 AI 的课程不同于大学英语课程，学生的专业背景对于这门课程的感知影响很大，比如英语只需要准备一些学生专业相关的视频、词汇等内容就实现了与他们专业知识的结合，但是计算机课程如果面对那些不需要使用计算机的专业，学生的积极性会比较低。但是同时也体现了学生缺乏终身学习和可持续发展的理念，只关注自己的专业领域。 | In English, preparing some major-related videos and vocabulary is enough to connect with students’ disciplines, but in a computer course, students from majors that do not use computers show low motivation. | Home-discipline repertoire does not transfer | C | Recorded practice or observation | A. Translation without a template |
| EA04 | W1 | R2:49,50 | C | 有部分学生会担心被 AI 取代，但是只是网络上笼统地听说过一些，并没有深入的了解AI 对于他们的专业到底有什么具体的影响。我通过一些课程内容的展示，介绍了 AI 对于他们这些专业的传统范式的影响，但是也提出了 AI 会创造新的更多的机会。鼓励他们创办自己的自媒体，学习使用 AI 工具，扩展自己的能力边界。但是学生的反应较为平淡。 有学生说到：现在的 AI 信息和新闻太多了，但是自己用过豆包，感觉并不聪明，那些绘图的AI 软件也试用过，感觉也一般。 | Some students worry about being replaced by AI, but only from vague things heard online… reactions were rather flat. A student said: ‘I have used Doubao and it doesn’t feel very smart.’ | Shallow AI awareness; low expectations | C | Recorded practice or observation | D. Uneven relevance and engagement |
| EA05 | W2 | R2:148 | C | 因为目前大学生对于网络的鉴别能力有限，经常会遇到虚假的下载信息和广告网站和非法欺诈网站，需要提高学生的鉴别能力。最常见的问题是，学生连一个 AI 应用的官网都没有找到，就打开了一个加的 AI 网站开始聊天。 | Students often opened a fake AI website and started chatting before they had even found the official site of the AI application. | Basic digital-literacy gap (online safety) | C | Recorded practice or observation | C. Drift to demonstration |
| EA06 | W2 | R2:170 | C | 体会到了计算机，包括 AI 应用的实践性很强。但是确实作为一门新的课程，缺少一些程序化的东西，比如英语当中经常要讲单词的用法， 在这门课中，暂时还不知道哪些是最基础的知识。只有在学生的实操环节才能看出来学生有哪些不会的部分。 | In English teaching we routinely explain how words are used, but in this course I do not yet know what the most basic knowledge is. I can only see what students cannot do during hands-on practice. | No curricular template; unclear ‘basics’ | C | Recorded practice or observation | A. Translation without a template |
| EA07 | W2 | R2:178 | C | 学生对于 AI 的了解并不多，也谈不上很关注，可能是因为他们刚刚从高中的高压环境当中毕业，进入“自由”的大学生活之后，更多的关注游戏和娱乐等内容。〔……〕学生对于 AI 学习的内驱力不足。问到他们对于 AI 的看法时，大部分学生还是低头不语或者看手机 | Most students kept their heads down in silence or looked at their phones. | Low participation | O | Recorded practice or observation | D. Uneven relevance and engagement |
| EA08 | W2 | R2:185 | C | 这次的课程需要很多的实操，但是学校的机房有很多的电脑损坏，更新的硬件要明年才能到位。给这次的实操课程增加了很多难度。只是通过 PPT 的宣讲很难实现效果，因为这些知识都是实际操作电脑的过程中才会遇到的问题。 | Many computers in the school’s lab are broken, and new hardware will not arrive until next year… explaining through slides alone can hardly work. | Hardware constraint | C | Recorded practice or observation | C. Drift to demonstration |
| EA09 | W2 | R2:192 | C | 增加一些网络视频的介绍，和自己的讲解示范结合起来，再增加学生的实操。 | Add some online videos, combine them with my own demonstration, and then add more hands-on practice. | Repair: video plus demonstration | M | Planned response (improvement section) | C. Drift to demonstration |
| EA10 | W3 | R2:221 | C | 学生的参与度一般，虽然表现出了对高级提示词的惊叹，但是具体操作时还是没有转化为适合自己应用场景的提示词。可能是因为这样一个看起来很简单的动作包含了较为高阶的思维方式的转化，不是简单的几个提示词展示就能教会学生的。另一个方面，一个或者一类提示词在给学生展示过之后，他们似乎就没有了继续自己操作一遍的动力，这个不同于电脑软件的操作，他们会觉得需要自己动手一遍才可以学会，提示词的使用，他们看过一遍就觉得自己已经了解了，这个不是很复杂的知识。或者是简单的把这些提示词方法收藏起来，后续需要使用的时候再查阅。 | They were amazed by advanced prompts but did not turn them into prompts for their own situations… once a prompt was shown, they seemed to lose the motivation to try it themselves… they feel they already understand after seeing it once… or they simply bookmark the methods. | Seeing-as-knowing | O | Recorded practice or observation | Thread: low-threshold illusion |
| EA11 | W3 | R2:236 | C | 使用英语作为 AI 的提示词确实会有更好的效果，但是这个效果的提升目前还是仅限于自己的使用，想让学生使用英语的提示词有些困难，这里毕竟不是英语课。更多的是向学生介绍提示词的思路。而这些思路的来源是英文的新闻和教程。逐渐让学生意识到英语对于获取最新 AI 知识的重要性。 | Using English prompts does give better results, but the benefit is limited to my own use; getting students to use English prompts is difficult—this is not an English class. I mostly introduced the thinking behind prompts, which comes from English-language news and tutorials. | Linguistic capital; linguistic scaffolding | M | Recorded practice or observation | B. Linguistic scaffolding |
| EA12 | W3 | R2:244 | C | AI 提示词是一个很难被传统学科分类的知识技能，它既不是“语文‘或者”英语“也不太像”计算机“的相关知识。更多的像是一种比这些学科还要底层的逻辑知识。但是提示词的使用又不足以成为一门独立的课程，所以目前来看，放在计算机、英语或者语文课堂上去介绍这些内容是比较合适的。 | Prompting is neither Chinese nor English, nor quite computing; it is more like a kind of logic beneath these subjects. | Prompting has no disciplinary home | C | Recorded practice or observation | B. Linguistic scaffolding |
| EA13 | W3 | R2:252,253 | C | 网络上关于 AI 提示词的内容其实很多，但是并不够系统，针对的是不同专业，不同层面的人群。而要将这些内容整合为大学课堂可以直接使用的材料其实需要进行重新的设计，包括 PPT 的制作等。其次，网络上这些内容的阅读者都是对于 AI 学习的积极性本身就比较高的群体，有些甚至是付费去主动学习相关的技能，而在大学课堂里面，教师还是需要花时间去介绍，去调动学生的积极性。 我是通过 AI 设计的较为美观的 PPT 和自己平时收集的看起来比较有趣、令人感觉意外的提示词效果来吸引学生眼球。 | Turning online content into classroom-ready material requires redesign… online readers are already motivated, whereas in class the teacher must spend time motivating students. I used attractive AI-designed slides and surprising prompt results to catch attention. | Redesign labour; attention-getting | M | Recorded practice or observation | A. Translation without a template |
| EA14 | W3 | R2:219,220 | C | 提示词的讲解例子，针对不同的专业，有不同的准备方案。 给学生布置的任务也是和专业相关的。 | Prompt examples were prepared differently for different majors, and assigned tasks were also major-related. | Major-specific examples | M | Recorded practice or observation | A. Translation without a template |
| EA15 | W4 | R2:342 | C | 本节课介绍 word 文档的编辑和制作，也涉及到了在AI 时代如何写文档，还有如何进行常见的格式排版。介绍了重要的理念，即格式与内容的分离。借助 AI 工具更快速的排版。 | Introduced an important idea: the separation of format and content; used AI tools to format faster. | Conceptual over procedural content | M | Recorded practice or observation | A. Translation without a template |
| EA16 | W4 | R2:373 | C | 艺术类学生目前用到word 文档的场景较少，大部分同学没有意识到文档编辑 和排版的重要性。 | Art students currently have few occasions to use Word; most did not realize the importance of document editing and layout. | Differential professional relevance | C | Recorded practice or observation | D. Uneven relevance and engagement |
| EA17 | W4 | R2:380,381 | C | AI 的更新比较快，具体到日常的使用，其实会涉及到很多工具，学校的电脑很多都没提前安装。系统自带的 word 版本又比较低。 通过一些在线的 markdown 转 word 工具进行展示，尽量避开本地工具。但是也使用了自己的电脑进行展示。 | Many lab computers did not have tools pre-installed, and the built-in Word version is old. I demonstrated with online Markdown-to-Word tools, avoiding local software, but also used my own computer. | Infrastructure workaround | M | Recorded practice or observation | C. Drift to demonstration |
| EA18 | W4 | R2:365 | C | 这一节课是融合 AI 与传统的 word 课程比较紧密的一节，即是计算机课，又是 AI 课程。这一部分我的短板主要是计算机部分的知识不够系统，特意复习了一下相关内容。 | My weakness is that my computing knowledge is not systematic; I specifically reviewed the relevant content. | Teacher self-upskilling | M | Recorded practice or observation | A. Translation without a template |
| EA19 | W5 | R2:466 | C | 本节课先介绍了传统的 PowerPoint 制作的一些基本知识，包括图层，文本框等基础知识。再过渡到现在常用的 WPS 模板套用来制作 PPT，最后再到通过 gamma，kimi 等 AI 软件直接生成 PPT，也介绍了通过 AI 生成 markdown 格式的 PPT 然后通过 marp 转为 PPT 格式。强调了提示词和提前设计的重要性，PPT 只是展示内容的一种方式，不能完全交给 AI。 | From traditional PowerPoint to WPS templates to AI generation (Gamma, Kimi) to Markdown with Marp… slides are only one way of presenting content and cannot be entirely handed over to AI. | Graduated traditional-to-AI sequence | M | Recorded practice or observation | A. Translation without a template |
| EA20 | W5 | R2:496 | C | 学生对 PPT 的重要性认识不足，虽然已经有些课程要求学生去做各种类型的汇报，但是他们的 PPT 制作也都比较简陋。他们存在的问题可能是过于依赖 AI 软件的一键 PPT 生成，缺乏自己的干预，最终导致 PPT 的展示效果不佳。 | They may rely too heavily on one-click slide generation in AI software, without their own intervention, which ultimately leads to poor presentations. | One-click generation without intervention | O | Recorded practice or observation | Thread: low-threshold illusion |
| EA21 | W5 | R2:503 | C | 设计吸引观众，吸引学生的 PPT 是一键比较困难的事情。现在有很多的短视频内容，很容易就夺走了学生的课堂注意力，导致他们对于 PPT 这种需要花更多精力去关注的内容失去耐心。更加缺少对制作 PPT 进行展示的兴趣。反而短视频的制作更加吸引学生。 | There is so much short-video content that it easily takes away students’ classroom attention, making them lose patience with slides… short-video production appeals to students more. | Attention competition (instructor hypothesis) | C | Recorded practice or observation | D. Uneven relevance and engagement |
| EA22 | W5 | R2:474 | C | 根据学生的专业，调整对应的示例。每个专业的班级进行展示的 PPT 不同。最终制作的随堂作业是他们的自我介绍，激发学生的积极性。 | The in-class assignment was a self-introduction, to spark students’ motivation. | Personally relevant task | M | Recorded practice or observation | A. Translation without a template |
| EA23 | W5 | R2:510 | C | PPT 的展示是一个综合技能，需要让学生上台展示。 | Presenting slides is a composite skill; students need to present on stage. | Generation is not communication | M | Planned response (improvement section) | E. Assessment and the visibility of learning |
| EA24 | W6 | R2:550 | C | 英语是我之前的专业教学科目，这次课进行起来比较轻松和熟悉。但是又没有真正的讲授英语的具体内容，而是从使用 AI 工具和方法的角度来介绍。需要更多的同理心，来体会学生对于英语学习的困难，而不是作为英语老师需要对学生提出很多要求的模式。 | English was my previous subject, so this class felt easy and familiar, yet I did not teach English content… I need more empathy for students’ difficulties, rather than the mode of an English teacher who places many demands on students. | Teacher reframing (turning point) | M | Recorded practice or observation | A. Translation without a template |
| EA25 | W6 | R2:558 | C | 学生对于英语学习的热情因专业不同和升学就业的前景区别而导致的差异很大，但是对于 AI 辅助英语学习的认知不是很充足，经常处于不借助 AI 和过分依赖 AI 的两个极端。 | Enthusiasm for English varies greatly by major and by study and employment prospects… students often sit at two extremes: not using AI at all or relying on it excessively. | Divided use (instructor observation) | O | Recorded practice or observation | D. Uneven relevance and engagement |
| EA26 | W6 | R2:528,536 | C | 借助 AI 提示词，来生成更符合学生专业特色的词汇和联系，实现英语的个性化学习。 根据学生的专业，调整对应的示例。每个专业的班级进行展示的 提示词不同，又的专业侧重口语的表达和训练，有的专业侧重于备考资料 | Using AI prompts to generate vocabulary and exercises matched to students’ majors… some majors emphasized oral expression, others exam preparation. | Major-specific personalization | M | Recorded practice or observation | B. Linguistic scaffolding |
| EA27 | W6 | R2:565 | C | 最大的挑战在于，很多高知〔sic，应为“高职”〕的学生对于英语的重要性认知不足，或者是自己的学生和专业工作的使用不多，因此内驱力不足。在介绍 AI 辅助英语学习的时候，也会遇到同样的问题。 | Many students do not recognize the importance of English… their intrinsic motivation is insufficient. | Perceived relevance of English | C | Recorded practice or observation | B. Linguistic scaffolding |
| EA28 | W6 | R2:572 | C | 更好的分层，面向不同需求的学生，进一步实现个性化的学习 | Better tiering for students with different needs. | Tiering (plan; not seen implemented) | M | Planned response (improvement section) | D. Uneven relevance and engagement |
| EA29 | W7 | R1:2617 | C | 卡片笔记法和 AI 赋能的第二大脑是网络上风靡一时的学习工具，但是现有的课程当中并没有。其中的理念性的东西很多，给学生介绍和理解需要一定的时间，但是课程安排时间又很有限。能感受到这个内容的重要性和有动力去学习的学生是少数。但是又是很前沿且很实用的 AI 应用，个人认为应该向学生介绍。 | These tools are popular online but absent from existing courses… only a minority of students sense their importance… but it is cutting-edge and practical, and I believe it should be introduced. | Teacher judgment on frontier content | M | Recorded practice or observation | A. Translation without a template |
| EA30 | W7 | R1:2603,2625 | C | 学生会感觉到神奇，但是又有点复杂。参与度不高，主要还是依赖教师讲授和视频展示。 学生感觉课程内容偏难，没有掌握，但是对于这些东西又有一定的好奇。希望可能通过更加简便的方式来实施这些先进的学习理念。 | Students find it amazing but somewhat complicated; participation was low, relying on lecturing and video. They felt it was difficult and did not master it, yet were curious and hoped for simpler ways. | Curious but unable to master | O | Recorded practice or observation | D. Uneven relevance and engagement |
| EA31 | W7 | R1:2632 | C | 学校的机房限制，这门课程提到的笔记和 AI 软件只能在教师机展示。 | Because of lab restrictions, the note-taking and AI software could only be demonstrated on the teacher’s computer. | Demonstration only (lab restriction) | C | Recorded practice or observation | C. Drift to demonstration |
| EA32 | W7 | R1:2639 | C | 使用国内一些整合比较好的，更加易用的软件来实现这个内容。 | Use more integrated, easier-to-use domestic software. | Lowering tool thresholds (plan) | M | Planned response (improvement section) | C. Drift to demonstration |
| EA33 | W8 | R8:3 | R | 这一周开始了 Excel 教学，说实话对他们来说有点难，简单的合并单元格，排序，对他们来说都很困难，必须一步一步的来展示、纠正才可以。我原本计划是然他们先练习传统的 Excel 操作，再通过 AI 控制 Excel，体验 AI 加速操作的过程，也能弥补一些 AI 操作不完善的地方。但是他们会选择直接 AI 操作，放弃传统的方式。我不知道这是真正的“AI 原生”还是只是懒惰； | Merging cells and sorting were very difficult; I had to demonstrate and correct step by step. I planned traditional Excel first, then AI control… but they chose to operate directly with AI and abandoned the traditional way. I do not know whether this is truly ‘AI-native’ or just laziness. | Bypassing foundational skills; instructor’s uncertain reading | O | Recorded practice or observation | Thread: low-threshold illusion |
| EA34 | W9 | R8:7 | R | 这个课是微课制作，我进行了全程的实机操作演示，从配图生成，音乐制作到视频剪辑。但是因为有一个班的出勤率已经明显降低，最终的教学效果收到影响。学生们对最终制作完成的视频还比较感兴趣，因为是根据当天的课堂活动，包括缺勤的状况来编辑的歌词进行创作的。但是也没法让学生完整的操作，因为流程较长，而且所需要的 AI 工具比较多，都是需要付费的，免费账户的额度不够。最终也只能停留在展示环节 | One class’s attendance had clearly dropped… the process is long and requires many AI tools, all paid; free accounts lack quota. In the end it could only remain at the demonstration stage. | Demonstration only (paywall/quota); attendance decline | C | Recorded practice or observation | C. Drift to demonstration |
| EA35 | W10 | R8:11 | R | 讲了 workflow 和 agent，也是只能展示为主，还给学生看了 B 站的一些视频。这是一个 AI 课程的矛盾，越是前沿的东西，越是只能展示。因为需要一定的硬件和付费账户，或者说算力的支持，另外之前 AI 的使用基础其实也是应用最新 AI 工具的一条隐形门槛。所以会有一种 AI 工具不需要上手门槛，每个人都很容易学到的错觉。实机操作下来，他们连API key 要往哪里粘贴都不知道。 | The more cutting-edge something is, the more it can only be demonstrated… prior AI experience is an invisible threshold… there is an illusion that AI tools require no entry threshold… they did not even know where to paste an API key. | Frontier–demonstration paradox; hidden threshold; perceived low threshold | C | Recorded practice or observation | Thread: low-threshold illusion |
| EA36 | W10 | R8:13 | R | 学期刚结束之后，这个 workflow 的课程就被我删掉了，换成了更新的更自动化的 agent 内容，AI 课程的内容更新太快了，我必须时刻保持学习，并且要最起码以学期为单位更新教学内容；新的 AI agent操作起来更方便了，但是可能依然会有那个实际操作的问题。 | Right after the semester ended, I removed this workflow unit and replaced it with newer, more automated agent content… I must update teaching content at least every semester. | Semester-scale redesign (post-semester) | M | Post-semester decision | C. Drift to demonstration |
| EA37 | W11 | R8:17 | R | 这一周展示了针对不同专业班级的一些 AI agent 应用，也是我从网上获取的最新资讯，最新的 AI 使用案例，作为一个比较有趣的环节加入到课堂中。比如使用生图功能给学生做穿搭、化妆的建议。学生的课堂参与度较高，课堂氛围良好，但是他们能从中收获的内容，我感觉并不多。 | Participation was high and the atmosphere good, but I feel they did not gain much from it. | Engagement is not learning | O | Recorded practice or observation | D. Uneven relevance and engagement |
| EA38 | W12 | R8:21 | R | 最后一次课，介绍考试的相关内容。进行提前预演。考试内容是另一个老师设计的，我进行的相同模式下的内容改变。其实就是根据一首中国古诗来进行生图和创作视频，原意是希望也能有一定的课程思政的教化作用，起码最终汇报的时候有这个感觉。因为全程可以在手机上操作，学生的完成度比较高，但是可以看出来，即便是这学期的课程一次都没上过，他们还是可以完成几乎一样的期末视频内容。虽然经过认真学习的同学最终交付的作品明显更好。但是我感觉这还是加重了他们对于 AI 门槛的幻觉。有的同学觉得，似乎没有那些技巧，做出来的东西也还可以凑合，学不学又有多大区别，反正认真做了别人也能看出来是 AI 制作的。 | The exam was designed by another teacher; I changed content within the same format: images and a video from a classical Chinese poem… it can be done entirely on a phone, and completion was high. | Externally designed assessment | C | Recorded practice or observation | E. Assessment and the visibility of learning |
| EA39 | W12 | R8:21 | R | 最后一次课，介绍考试的相关内容。进行提前预演。考试内容是另一个老师设计的，我进行的相同模式下的内容改变。其实就是根据一首中国古诗来进行生图和创作视频，原意是希望也能有一定的课程思政的教化作用，起码最终汇报的时候有这个感觉。因为全程可以在手机上操作，学生的完成度比较高，但是可以看出来，即便是这学期的课程一次都没上过，他们还是可以完成几乎一样的期末视频内容。虽然经过认真学习的同学最终交付的作品明显更好。但是我感觉这还是加重了他们对于 AI 门槛的幻觉。有的同学觉得，似乎没有那些技巧，做出来的东西也还可以凑合，学不学又有多大区别，反正认真做了别人也能看出来是 AI 制作的。 | Even students who had not attended a single class could produce almost the same final video, although the work of students who studied seriously was clearly better. | Assessment does not discriminate learning | O | Recorded practice or observation | E. Assessment and the visibility of learning |
| EA40 | W12 | R8:21 | R | 最后一次课，介绍考试的相关内容。进行提前预演。考试内容是另一个老师设计的，我进行的相同模式下的内容改变。其实就是根据一首中国古诗来进行生图和创作视频，原意是希望也能有一定的课程思政的教化作用，起码最终汇报的时候有这个感觉。因为全程可以在手机上操作，学生的完成度比较高，但是可以看出来，即便是这学期的课程一次都没上过，他们还是可以完成几乎一样的期末视频内容。虽然经过认真学习的同学最终交付的作品明显更好。但是我感觉这还是加重了他们对于 AI 门槛的幻觉。有的同学觉得，似乎没有那些技巧，做出来的东西也还可以凑合，学不学又有多大区别，反正认真做了别人也能看出来是 AI 制作的。 | This deepened their illusion about AI’s threshold… so what difference does learning make; anyway, others can tell it was made with AI. | Low-threshold illusion reinforced; learning devalued | O | Recorded practice or observation | Thread: low-threshold illusion |

Counts — anchors: 40; strata: {‘C’: 32, ‘R’: 8}; CMO: {‘M’: 17, ‘C’: 14, ‘O’: 9}; primary theme: {‘A’: 11, ‘D’: 9, ‘C’: 8, ‘Thread’: 5, ‘B’: 4, ‘E’: 3}.
